# Effect of a qigong intervention program on telomerase activity and psychological stress in abused Chinese women: a randomized, wait-list controlled trial

**DOI:** 10.1186/1472-6882-14-300

**Published:** 2014-08-15

**Authors:** Agnes Tiwari, Cecilia Lai Wan Chan, Rainbow Tin Hung Ho, George Sai Wah Tsao, Wen Deng, Athena Wai Lin Hong, Daniel Yee Tak Fong, Helina Yin King Yuk Fung, Emily Pei Shin Pang, Denise Shuk Ting Cheung, Joyce Lai Chong Ma

**Affiliations:** School of Nursing, Li Ka Shing Faculty of Medicine, The University of Hong Kong, 4/F, William M.W. Mong Block, 21 Sassoon Road, Pokfulam, Hong Kong; Department of Social Work and Social Administration, The University of Hong Kong, The Centennial Campus, The University of Hong Kong, Room 534, Jockey Club Tower, Pokfulam, Hong Kong; Department of Anatomy, Li Ka Shing Faculty of Medicine, The University of Hong Kong, Faculty of Medicine Building, 21 Sassoon Road, Pokfulam, Hong Kong; HKSKH Lady MacLehose Centre, No.22, Wo Yi Hop Road, Kwai Chung, New Territories Hong Kong; Department of Social Work, The Chinese University of Hong Kong, Level 4&5, T.C. Cheng Building, United College, Shatin, New Territories Hong Kong

**Keywords:** Qigong, Violence, Telomerase, Perceived stress, Abused women, Intimate partner violence (IPV), Intervention, Depression, Chinese, Randomized controlled trial (RCT)

## Abstract

**Background:**

Abused women, who suffer from chronic psychological stress, have been shown to have shorter telomeres than never abused women. Telomere shortening is associated with increased risk of cell death, and it is believed that adopting health-promoting behaviors can help to increase the activity of telomerase, an enzyme that counters telomere shortening. Qigong is an ancient Chinese mind-body integration, health-oriented practice designed to enhance the function of *qi*, an energy that sustains well-being. Therefore, an assessor-blind, randomized, wait-list controlled trial was developed to evaluate the effect of a qigong intervention on telomerase activity (primary objective) and proinflammatory cytokines, perceived stress, perceived coping, and depressive symptoms (secondary objectives) in abused Chinese women.

**Methods/Design:**

A total of 240 Chinese women, aged ≥18 years, who have been abused by an intimate partner within the past three years will be recruited from a community setting in Hong Kong and randomized to receive either a qigong intervention or wait-list control condition as follows: the qigong intervention will comprise (i) a 2-hour group qigong training session twice a week for 6 weeks, (ii) a 1-hour follow-up group qigong exercise session once a week for 4 months, and (iii) a 30-minute self-practice qigong exercise session once a day for 5.5 months. The wait-list control group will receive qigong training after the intervention group completes the program. Upon completion of the qigong intervention program, it is expected that abused Chinese women in the intervention group will have higher levels of telomerase activity and perceived coping and lower levels of proinflammatory cytokines, perceived stress, and depressive symptoms than will abused Chinese women in the wait-list control group.

**Discussion:**

This study will provide information about the effect of qigong exercise on telomerase activity and chronic psychological stress in abused Chinese women. The findings will inform the design of interventions to relieve the effects of IPV-related psychological stress on health. Also, the concept that health-promoting behaviors could slow down cellular aging might even motivate abused women to change their lifestyles.

**Trial registration:**

Current Controlled Trials NCT02060123. Registered February 6, 2014.

## Background

Intimate partner violence (IPV), also known as domestic violence, involves assaultive and coercive power and control tactics used by a current or former partner to cause emotional, physical, and/or sexual harm [1]. While both men and women can be victims of IPV, most victims are women [2]. Moreover, the female partner receives the majority of IPV-related injuries [2].

IPV is a global problem. For example, the World Health Organization estimates that from 10% to 50% of women worldwide have been physically or sexually assaulted by their intimate partners at some time in their lives [3]. Additionally, overwhelming evidence indicates that IPV is linked to adverse health outcomes [4, 5] and that it is a greater health burden than smoking and obesity [6].

Despite the abundant evidence linking IPV to negative health outcomes [4], the mechanisms underlying this association are not fully understood[7]. It is often assumed that the greater morbidity experienced by abused women is due to the chronic psychological stress of having to live in an atmosphere of fear, shame, and coercion [5]. More specifically, a recent study suggests that IPV-related stress may accelerate cellular aging, which in turn may attenuate normal bodily functions and lead to greater morbidity [8].

Cell growth is essential to the continuity of living organisms. Cellular senescence or aging is a state of permanent cell growth arrest that is believed to be a damage response to a variety of stressors [9]. Excessive accumulation of senescent cells may lead to organ dysfunction. One of the basic mechanisms by which normal somatic cells enter into senescence involves continuous shortening of a chromosomal terminal structure called the telomere. Telomeres are nucleoprotein structures at the ends of chromosomes, and their function is to protect the chromosome ends from deterioration or fusion with other chromosomes. Telomeres generally shorten with aging, but the shortening may be accelerated in the presence of chronic psychological stress [10]. A cellular enzyme termed telomerase elongates telomeres by adding DNA hexameric repeats to restore the length of the telomere [11]. Accelerated telomere shortening occurs in cells with lowered telomerase activity and in those under oxidative stress. Accelerated telomere shortening may be a predictor of early morbidity and mortality [8].Thus, telomeres and telomerase play prominent roles in maintaining normal cell function and genome integrity [11, 12].

Various forms of chronic psychological stress reportedly lead to telomere shortening. In one study, the perceived level of psychological stress in mothers who took care of children with chronic illness was associated with shorter telomeres, higher oxidative stress levels, and lower telomerase activity levels [10]. This association between chronic psychological stress and accelerated telomere shortening has been also observed in individuals with mood disorders including major depression, bipolar depressive disorder, and anxiety disorder [13–17] and in caregivers of patients with Alzheimer’s disease [18]. Study participants with a history of childhood maltreatment or other adversities have exhibited shorter telomeres than those without such experiences [16, 19]. In another study, women with a history of intimate partner abuse had shorter telomeres than did never abused women [8].

Several researchers have suggested that telomerase activity may be increased by health-promoting behaviors such as physical exercise [20, 21], intensive meditation training [22, 23],yogic meditation[24, 25], and comprehensive lifestyle changes (including regular physical exercise and psychological stress counseling) [26]. More recently, Chinese people with chronic fatigue or chronic fatigue syndrome were shown to have higher telomerase activity levels after undergoing qigong exercise intervention than did the control group, supporting the speculation that qigong may enhance telomerase activity by promoting mindfulness and managing stress [27].

### Health benefits of qigong

Qigong refers to the cultivation or enhancement of *qi*. According to Traditional Chinese Medicine, *qi* is an energy that sustains human well-being and assists in healing [28]. Qigong originates from ancient healing and medical practices in Asia and dates back more than 5000 years. It comprises a series of orchestrated practices including body postures such as standing or sitting, the performance of a range of simple movements, breath practice to accompany the postures or movements, and meditation to achieve a focused state of relaxed awareness. All of these practices are designed to enhance the function of *qi* through the attainment of deeply focused and relaxed states [28]. Many forms of qigong have been developed over the centuries. Some are designed for spiritual practice, while others are designed to enhance martial art skills. Still others were designed for general health enhancement, including the Eight Brocade (or *baduanjin*), which is the form of qigong reported in this paper. By evoking body–mind integration, health-oriented qigong practices (including the Eight Brocade) are thought to activate self-healing by stimulating the balanced release of endogenous neurohormones and other natural health recovery mechanisms [28].

In a systematic review of randomized controlled trials examining the health benefits of qigong, anxiety was found to be significantly lower among participants practicing qigong than among those in the control group engaged in active exercise [29–31]. The severity of depression was also significantly lower after qigong than in an inactive control group engaged in newspaper reading [32]. However, non-statistically significant changes in depression were found when qigong was compared with a control group of subjects who underwent no qigong training during the same study period [33] and with a control group engaged in exercise [29, 34]. Significantly lower norepinephrine, epinephrine, and cortisol blood levels were found in the qigong group than in a wait-listed control group [35]. Qigong was also shown to improve chronic fatigue and increase telomerase activity in a study of Chinese patients, as described earlier [27]. Taken together, the above findings suggest that qigong may have beneficial health effects. Specifically, the protective effect of qigong as a mind–body intervention on telomere and telomerase activity should be further investigated. If the meditation component of qigong protects telomeres or telomerase activity by reducing chronic psychological stress, as has been suggested for other meditation training [36], we would expect to find that qigong intervention also has protective effects on telomere or telomerase activity in people experiencing chronic psychological stress. IPV has long been recognized as a potent source of chronic psychological stress [37]. An earlier study revealed that abused women had significantly shorter telomeres than did never-abused women [8], suggesting a link among IPV, chronic psychological stress, and accelerated cellular aging. However, evidence on the optimal telomere or telomerase activity protection measure in abused women is still lacking.

This study protocol describes a randomized controlled trial that evaluates the effect of a qigong intervention on telomerase activity and other parameters in abused Chinese women. The decision to use qigong in this study is based on the findings of our earlier intervention [38] in which Chinese medicine dietary regimens based on individual health assessments by a Chinese medicine practitioner were provided to abused women in a shelter in Hong Kong. An imbalance and disharmony of *qi* was found to be the root cause of the women’s health symptoms, the most common being insomnia, headache, palpitation, and fatigue. Furthermore, the participants who adhered to the intervention, namely the individualized Chinese medicine dietary regimen, reported significantly greater improvement in their health symptoms [38] than did participants who did not adhere to the regimen. These results indicate that the Chinese abused women were receptive to engaging in health-promoting behaviors based on the concepts of Chinese medicine and benefited from an intervention that promoted the balance and harmony of *qi*. We have therefore decided to adopt qigong as an intervention to promote abused Chinese women’s health and well-being.

### Aims

The primary aim of this randomized, wait-list controlled trial is to evaluate the effect of a qigong intervention on telomerase activity in Chinese women with a history of IPV. The secondary aim is to evaluate the effect of the intervention on the women’s levels of proinflammatory cytokines, perceived stress, perceived coping, and depressive symptoms.

We hypothesize that upon completion of the qigong intervention, abused Chinese women in the intervention group will have higher telomerase levels as measured by telomerase activity in peripheral blood mononuclear cells, lower proinflammatory cytokine levels as measured by tumor necrosis factor-alpha (TNF-alpha) and interleukin-6 (IL-6) in peripheral blood mononuclear cells, lower perceived stress scores as measured by the Perceived Stress Scale, higher perceived coping scores as measured by the Perceived Coping Scale, and lower depressive symptoms scores as measured by the Beck Depression Inventory version II than will women in the wait-list control group.

## Methods/Design

This randomized, wait-list controlled study will comprise two arms: an intervention group that will undergo immediate treatment (qigong intervention) and a wait-list control group that will undergo delayed treatment (the same qigong intervention, but provided after the intervention group has completed the immediate treatment). Thus, this randomized, wait-list controlled design will allow all study participants to eventually undergo the intervention while also controlling for variables that could cause spurious causality. The justification for using a wait-list control group in this study is that (i) the anticipation of undergoing qigong training in the near future can act as an incentive for the control group to participate in the study and (ii) qigong can be provided to both groups without increasing the risk of treatment contamination.

### Participants

Chinese women will be eligible to participate if they are:

- ≥18 years of age,
- willing to undergo the qigong intervention,
- available for all testing points,
- receptive to random allocation, and
- confirmed to have been abused by an intimate partner within the past three years based on the Chinese Abuse Assessment Screen (C-AAS).

Chinese women will be excluded if they:

- have participated in qigong training or other mind–body interventions within the previous 6 months,
- have serious medical conditions that might limit their participation in qigong exercise (based on our previous experience, such conditions include cancer, severe obesity, narcolepsy, major depressive disorder, and schizophrenia),
- have psychiatric disorders,
- use medication or other psychological intervention for stress, or
- have been abused by someone other than their intimate partner.

### Sample size

Sample size calculation will be based on a primary comparison of telomerase activity levels between the intervention and control groups. Based on a previous study that examined the effect of qigong on telomerase activity [27], the standard deviation will be set at 0.2. As in the study by Ho et al. we anticipate approximately 0.05 to 0.10 units of improvement in telomerase activity secondary to qigong. Thus, 113 subjects per group are required to detect a difference of at least 0.075 units between the two groups with 80% power and a ≤5% chance of a false-positive error. Allowing for a small rate of attrition, 240 women will be recruited and randomly assigned to either the intervention group (treatment condition, n = 120) or the wait-list control group (control condition, n = 120).

### Setting

This trial will be conducted in a nongovernment organization (NGO) in Hong Kong. The NGO has a large catchment area covering three districts in Hong Kong with >500,000 inhabitants. Annual official statistics indicate that these 3 districts have consistently higher rates of IPV among all 18 districts in Hong Kong [39].

### Recruitment

We will adopt the same recruitment strategies successfully used in our previous trials in the same community [40]. These recruitment strategies include conducting promotional sessions in the host NGO community center and its numerous outreach sites, providing recruitment talks in the neighborhood public housing estates where IPV is prevalent, enlisting the help of school principals to facilitate subject recruitment from among the mothers of the students, recruiting subjects from those participating in the well-established community networks, and advertising this project in the newsletters published by the host NGO.

For recruitment, a face-to-face meeting will be arranged in a private area with each potential participant. Following a well-established protocol to ensure the woman’s safety and consistency of the recruitment procedures [40], one of our trained research assistants will explain the trial to the woman, including the nature of a randomized, wait-list controlled trial; her rights as a research subject; and the need for obtaining blood specimens from her. If she agrees, she will sign a written consent form. The C-AAS will then be administered. Women determined to be “not abused” according to the C-AAS results will be thanked for their interest and assured that no further contact will be made. Those determined to be “abused” and who meet the inclusion criteria will be enrolled in the study.

### Randomization and blinding

All eligible participants will be randomized to either the intervention or wait-list control group according to a list of random permutations prepared by block randomization performed by a person not involved in subject recruitment. The block size will be known only by the randomizer. The order of the allocation, which will not be altered, will be centrally controlled to avoid any bias in selection. The research assistants will call the central-control center to obtain the list of random permutations for the allocation.

The questionnaires will be self-completed by the women and numerically coded without revealing the group allocation of the participants. A list of the numerical codes and names of the participants will be kept separately and securely in the central-control center. The researchers conducting the measurement of telomerase activity and its modulating biochemical factors will be blinded to the group allocation.

### Intervention

The qigong intervention in this project is based on the intervention tested by our team members (Chan, Ho) in an earlier study that involved participants with chronic fatigue and achieved positive outcomes [27]. The intervention comprises (i) group learning and practice: a 2-hour qigong exercise training session provided by a qigong master twice a week for 6 consecutive weeks (total, 24 hours), (ii) weekly group follow-up: a 1-hour qigong exercise session provided by a qigong master once a week for 4 consecutive months (total, 16 hours) after the group learning and practice, and (iii) self-practice: a 30-minute qigong exercise session performed by each individual participant once a day for the whole intervention period (total, 63 hours). Each participant will perform a total of 103 hours of qigong during the 5.5-month intervention period.

For each of the 2-hour qigong exercise training sessions during the 6-week group learning and practice periods, the activities will include (i) a brief introduction to the basic theories of traditional Chinese medicine and the physiology of mind–body connections, (ii) gentle movement or body stretching in a standing posture to facilitate a harmonious flow of *qi* along the energy channels, and (iii) qigong exercise training delivered by an experienced qigong master.

### Procedures

Upon entry into the study (T0, baseline), women in both groups will be asked to complete the Chinese version of the Revised Conflict Tactics Scales (CTS2), Perceived Stress Scale (PSS), Perceived Coping Scale (PCS), and Beck Depression Inventory version II (BDI-II) as well as a demographic questionnaire. Blood samples will be collected from each of the participants by a research nurse trained for this purpose. The intervention group will then receive the qigong intervention. To monitor the self-practice of qigong by participants in the intervention group, we will adopt the recording and monitoring process that was successfully used in the earlier qigong intervention study conducted by our team members [27]. Specifically, each participant will enter on a record card the details of when she practices qigong, for how long, and how many times each day, together with the type and duration of other exercises undertaken in the same day. The participant will be required to submit the record card to our researcher to check the self-practice compliance at the weekly group follow-up sessions. The weekly monitoring will allow the researchers to undertake timely measures to address any early signs of noncompliance. Participants in the wait-list control group will be advised to avoid joining any outside qigong training classes because qigong exercise training will be provided to them after the final outcome measurement. Additionally, to reduce the risk of attrition, the control group will receive monthly health education sessions unrelated to qigong, commencing at the point at which the intervention group starts the weekly group follow-up.

When the intervention group begins the post-training and postintervention periods (T1 and T2, respectively), participants in both groups will complete the CTS2, PSS, PCS, and BDI-II. Blood specimens will be collected from the participants in both groups at T2. When the intervention group has completed the qigong intervention, participants in the wait-list control group will receive the qigong exercise training.

**Figure 1:**
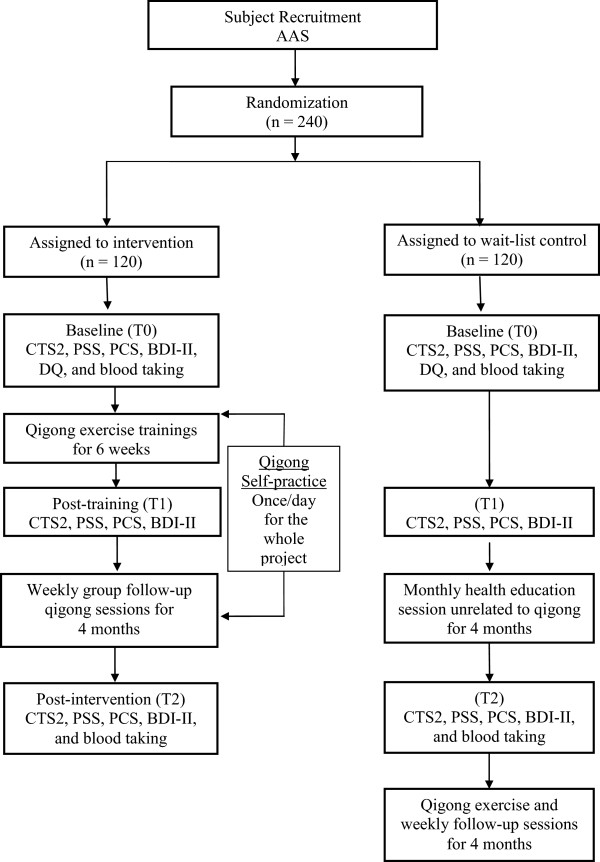
**Flow diagram of intervention/wait-list control and data collection points.**

The immediate treatment received by the intervention group, the wait-list control condition received by the control group, and the data collection points is shown in Figure 1.

Maintaining minimal attrition throughout the 5.5-month period will be important. Therefore, we intend to use the same field tracking strategies that we used in our previous project [40], which achieved zero attrition. Specifically, we will use systematic field tracking strategies that model the systemized tracking methodology used by McFarlane et al. to achieve a high retention rate [41]. Details of the tracking strategies, which have proven effective in providing early warnings about possible drop-outs, were previously reported [40].

Furthermore, we shall implement the following additional measures to reduce attrition and enhance the participants’ adherence to the intervention:

- Weekly group follow-up sessions provided by a qigong master to reinforce learning and remedial teaching and motivate the participants to engage in learning and practice
- Formation of small subgroups to promote group cohesion and support
- Election of a peer leader of each subgroup to enhance group communication and sharing
- Recording of qigong and other exercises undertaken on a record card, which will be submitted to our researcher at the weekly group follow-up sessions to check each participants’ self-practice compliance
- Monthly health education talks unrelated to the qigong intervention for participants in the wait-list control group
- All participants will be informed at the time of recruitment and regularly throughout the study that upon successful completion of the project, they will receive a health report based on the data collected as well as a DVD and book on *baduanjin.*

Finally, we will provide an incentive to the participants in the form of food coupons to show our appreciation of their time and efforts.

### Measurements

#### Instruments

The following study instruments with the exception of the C-AAS and demographic questionnaire will be administered at three time points: (a) preintervention (T0, baseline); i.e., upon entry into the study, after randomization but before intervention; (b) post-training (T1, 6 weeks later); i.e., upon completion of the 6-week group learning and practice session; and (c) postintervention (T2, 5.5 months later); i.e., upon completion of the entire qigong intervention program. The C-AAS and demographic questionnaire will only be administered at baseline.

- The five-item *C-AAS*, which has demonstrated satisfactory psychometric properties [42], will be used to screen potential participants for IPV. If a woman answers “yes” to being emotionally, physically, or sexually abused within the past three years and if the perpetrator is her former or current intimate partner, she is considered to be positive for IPV.
- The *CTS2*, which has been validated with satisfactory validity and reliability [43], will be used to measure the type and frequency of behaviors used by the perpetrator during partner conflict. This 27-item instrument contains 8 measures of psychological aggression, 12 measures of physical assault, and 7 measures of sexual coercion. How often each behavior occurred within the preceding year is indicated by 0 = not within the past year, 1 = once, 2 = twice, 3 = 3 to 5 times, 4 = 6 to 10 times, 5 = 11 to 20 times, and 6 = 21 or more times.
- The 10-item *PSS*, the most widely used psychological instrument for measuring the perception of stress [44], will be used to assess the degree to which situations in life have been perceived by the participant as stressful during the past month. The PSS contains six negative and four positive items, and the overall score is obtained by reversing the responses to the four positive items (i.e., 0 [never] = 4, 1 [almost never] = 3, 2 [sometimes] = 2, 3 [fairly often] = 1, and 4 [very often] = 0) and then summing the response scores across all scale items. The PSS score may range from 0 to 40.
- The *PCS* [45] will be used to assess the types and perceived effectiveness of each of the 13 specific strategies used by the participant in dealing with violence perpetrated by her intimate partner. The strategies may be classified as active or passive; e.g., seeking assistance from family or friends, confronting the partner, redefining the meaning of the situation, or using alcohol or drugs. Scoring is based on a four-point Likert scale: 1 (not at all helpful), 2 (not so helpful), 3 (somewhat helpful), and 4 (very helpful).
- The Chinese version of the *BDI-II* will be used to assess depressive symptoms experienced within the previous 2 weeks. This 21-item instrument has established construct validity and reliability for Chinese populations [46]. The score may range from 0 to 63, with 0 through 13 indicating minimal depressive symptoms, 14 through 19 indicating mild depressive symptoms, 20 through 28 indicating moderate depressive symptoms, and 29 through 63 indicating severe depressive symptoms.
- A *demographic questionnaire* will be used to elicit the following information: age of the participant and her partner, education level, place of birth, number of years living in Hong Kong, marital status, duration of the abusive relationship, number of children, employment status of the participant and her partner, financial hardship, receipt of comprehensive social security assistance, need for financial support, and history of chronic illness.

#### Measurement of telomerase activity and its modulating biochemical factors

Previous studies have shown that significant chronic stress-induced changes in telomere lengths cannot be detected over a short period of time, such as 1 to 2 years [26] and that telomerase has multiple telomere-independent and stress-resistant functions [47]. Thus, measurement of the changes in telomerase activity is an optimal alternative and has been successfully performed [26]. The biochemical modulators of telomerase activity include proinflammatory cytokines such as TNF-alpha and IL-6 [48], which are closely related to chronic psychological stress. Elevated levels of inflammation have been observed in IPV survivors [37]. We anticipate that qigong exercise will result in increased telomerase activity, which is associated with decreased levels of the proinflammatory cytokines TNF-alpha and IL-6 in IPV survivors. For each participant in this proposed study, 10 ml of peripheral blood will be collected to measure telomerase activity and TNF-alpha and IL-6 levels at preintervention (T0) and postintervention (T2)in the intervention group as well as before qigong training in the wait-list control group. Peripheral blood mononuclear cells will be isolated for telomerase activity measurement. Telomerase activity will be analyzed using a commercially available kit (*T*elo*TAGGG* Telomerase PCR ELISA; Roche) as previously used by our team members [27]. This method allows for highly specific amplification of telomerase-mediated elongation of products combined with nonradioactive detection following an ELISA protocol. Because this method is PCR-based, it provides a way to perform highly sensitive and quantitative assays of telomerase activities for a large number of samples. This kit has been used in many laboratories worldwide for several years and has resulted in numerous publications, including those in high-impact journals (e.g., Agarwal et al. *Nature* [49]; Strååt K et al. *Journal of the National Cancer Institute* [50]; Greco et al*. Stem Cells* [51]; Ball et al. *Aging Cell* [52]). Our research team members (Chan, Ho) have successfully used this telomerase measurement method to detect significantly greater telomerase activity in mononuclear cells of subjects with chronic fatigue after 4 months of qigong practice than in the control group [27].Finally, the plasma levels of the proinflammatory cytokines TNF-alpha and IL-6 will be measured using commercially available ELISA kits (R&D Systems).

### Data analysis

The primary outcome in this study will be the telomerase level as measured by telomerase activity at postintervention (T2). The secondary outcomes will be the proinflammatory cytokines TNF-alpha and IL-6, perceived stress as measured by the PSS, perceived coping as measured by the PCS, and depressive symptoms as measured by the BDI-II at postintervention (T2).

Data analysis will be performed using Statistical Analysis System software. Each estimated effect will be accompanied by a 95% confidence interval where appropriate. Unless otherwise specified, a 5% level of significance will be used. All statistical methods will be checked for validity before interpretations are made.

The baseline (T0) characteristics between the intervention and wait-list control groups will be assessed by a chi-squared test and Mann–Whitney U test for categorical and continuous data, respectively.

#### Primary analysis

The telomerase activity upon completion of the intervention (i.e., T2) between the intervention and wait-list control groups will be assessed by regression analysis with adjustment of baseline values. Residuals will be checked to ensure the adequacy of the method. Additionally, the change in telomerase activity from baseline (T0) to postintervention (T2) will be assessed by a paired t-test. The intention-to-treat principle will be adopted, and all study subjects will be included in the analysis. Missing values will be replaced by the last observed values.

#### Secondary analysis

Differences in the levels of the proinflammatory cytokines TNF-alpha and IL-6 and differences in the PSS, PCS, and BDI-II scores will be compared between the intervention and wait-list control groups by a linear mixed-effects model. The baseline values of the scale and intervention group will serve as fixed factors, and the intercept will serve as a random factor. Moreover, the effects of demographics on the outcomes will be explored by considering them as fixed factors in the linear mixed-effects model. Finally, changes from baseline will be assessed by a linear mixed-effects model using linear contrasts.

### Ethical issues

This protocol was approved by the Institutional Review Board of the University of Hong Kong/Hospital Authority Hong Kong West Cluster (HKU/HA HKW IRB: UW 12–555) on 9 January 2013.The study will be conducted according to the Declaration of Helsinki. Participation in the study is voluntary. An information sheet will be provided and a written consent is required from all participants.

In our extensive experience of working with abused women, we have found that they generally appreciate explaining their experience of abuse to professionals who have been trained to validate this experience. In this study, they will also be offered an opportunity for follow-up with a social worker if necessary. If they choose to withdraw from the study, they may do so with no questions asked.

## Discussion

This study protocol provides a detailed account of the implementation and evaluation of a qigong intervention for abused Chinese women. This will be the first randomized controlled trial to evaluate the effect of qigong on the telomerase activity and psychological stress levels of Chinese women who experience IPV.

The findings of this study will not only provide new knowledge supporting the design of other interventions to buffer the effects of IPV-related psychological stress on health, but they may also support the idea that health behaviors, such as qigong, can counter accelerated cellular aging and thus motivate abused women to change their lifestyles. These trial findings may also motivate professionals to provide timely interventions that interrupt declines in patient health and reduce suffering among abused women with the potential to lower IPV-related health costs.

This study will be conducted in Hong Kong in a group of community-dwelling Chinese women whose experience of IPV may or may not have been previously brought to the attention of social services or health professionals. This qigong intervention adds to the list of psychosocial interventions already available to abused Chinese women, including (i) a 30-minute session of empowerment intervention provided once by an antenatal clinic [53], (ii) a 3-week intervention providing empowerment training, legal advocacy, parenting training, and a Chinese medicine dietary regimen in a shelter setting [38],and (iii) a 12-week empowerment intervention with social support in a community setting [40]. This qigong intervention differs from the above-listed interventions by engaging women in health behaviors that empower them to better manage IPV-related psychological stress and hopefully attenuate the associated adverse health effects. As such, the results of this qigong intervention may expand the scope of interventions for abused Chinese women.
